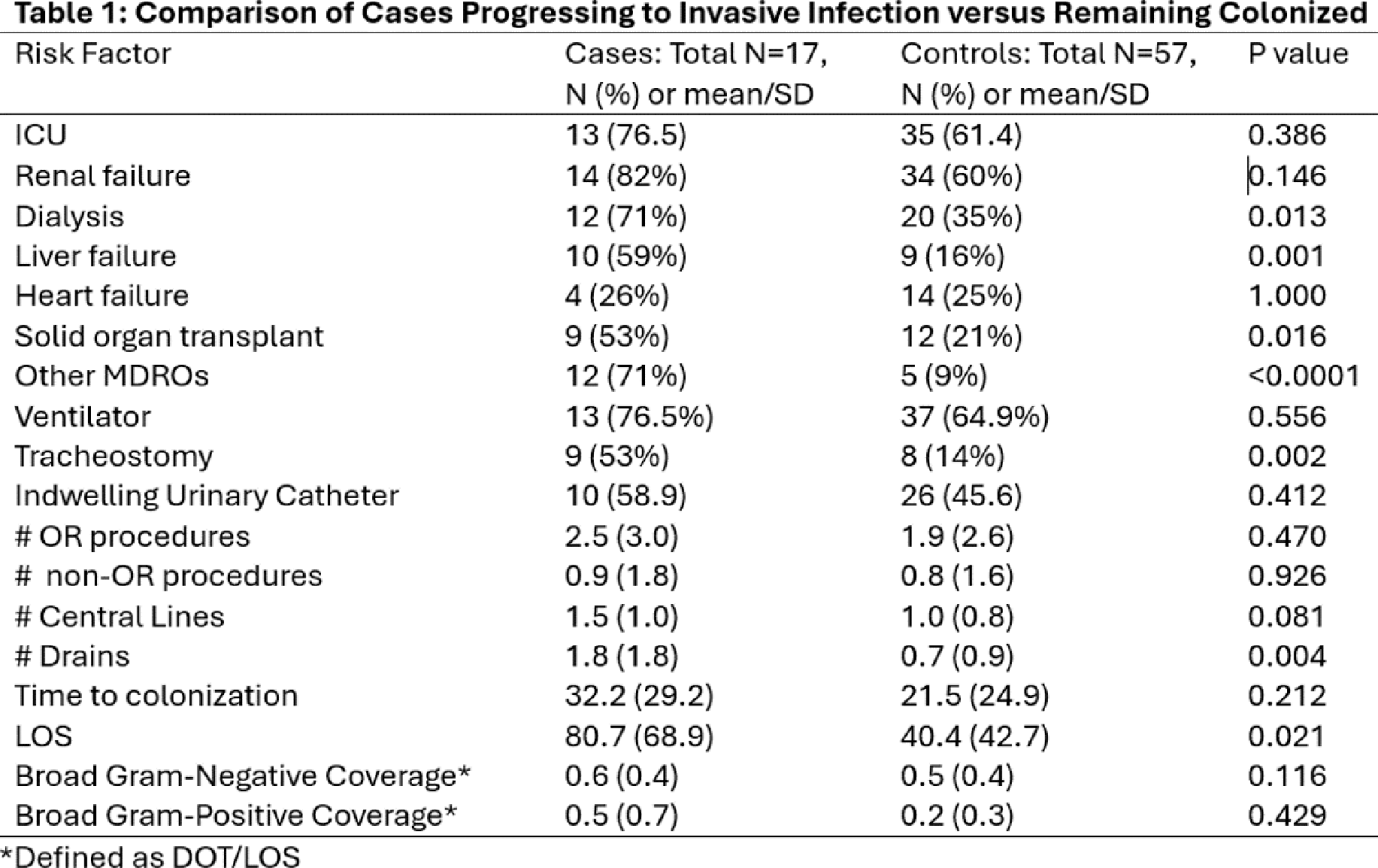# 247 Getting Ready for the Storm: A Taskforce Approach to Measles Readiness

**DOI:** 10.1017/ash.2026.10731

**Published:** 2026-06-23

**Authors:** Sonia Srikanth, Patrick Ching, Barry Rittmann, Yvette Major, Connie Atkinson, Jenna Price, Alexandra Bryson, Christopher Doern, Kaila Cooper, Michelle Doll

**Affiliations:** 1 VCU School of Medicine; 2 Virginia Commonwealth University; 3 VCUHS; 4 Children’s Hospital of Richmond at VCU Health; 5 VCU Health Healthcare Infection Prevention Program; 6 Nursing VCU Health

## Abstract

**Background:** Identified risk factors for Candida auris infection are generally markers of disease severity and/or medical vulnerability. A regional C. auris outbreak in 2022 led to extensive admission and point-prevalence testing in tertiary care hospital. We retrospectively reviewed charts to identify novel risk factors for progression from colonization to infection in a universally medically complex population. **Methods:** A case-control study was performed to compare patients developing invasive infection (cases) from those remaining colonization. Inclusion criteria were patients newly identified to be colonized with C. auris during a hospital admission from 1/2023 through 5/2025. Patients diagnosed with clinical infection as their first evidence of colonization were excluded. Risk factor variables included ICU status, other drug resistant organisms (MDROs), length of stay (LOS), time to colonization (days from admission to identification of C. auris), antimicrobial coverage with broadly acting agents, operative and other procedures, type/number of devices. Factors were compared between cases and controls using Fisher’s Exact Tests for categorical variables and Mann-Whitney U Tests for continuous variables using SAS 9.4. **Results:** During the study, 74 patients became colonized and 17 went on to develop invasive infection. The following were associated with infection: colonization with other MDROs, tracheostomy, liver failure, dialysis (though not renal failure), transplant w/in last year (driven by liver, N=7), number of drains at colonization (table 1). Several factors associated with severity of illness (ICU status, antibiotic pressure) were not associated with progression to infection in this cohort. Discussion: In a medically complex population, several risk factors associated with illness severity were not associated with progression from C. auris colonization to infection, whereas liver disease and transplantation, along with other MDROs, tracheostomy, and number of drains were. Targeted interventions attempting to decolonize tracheostomy or drain sites, and/or liver disease patients in general may help prioritize infection prevention where it is most needed.

**Figure:**